# The E3 Ubiquitin Ligase Cbl-b Predicts Favorable Prognosis in Breast Cancer

**DOI:** 10.3389/fonc.2020.00695

**Published:** 2020-05-05

**Authors:** Xiuming Liu, Yuee Teng, Xin Wu, Zhi Li, Bowen Bao, Yunpeng Liu, Xiujuan Qu, Lingyun Zhang

**Affiliations:** ^1^Department of Medical Oncology, The First Hospital of China Medical University, China Medical University, Shenyang, China; ^2^Key Laboratory of Anticancer Drugs and Biotherapy of Liaoning Province, The First Hospital of China Medical University, Shenyang, China; ^3^Liaoning Province Clinical Research Center for Cancer, Shenyang, China

**Keywords:** Cbl-b, breast cancer, overall survival, disease-free survival, prognosis, nomogram

## Abstract

**Background:** Casitas B-lineage lymphoma proto-oncogene-b (Cbl-b) is an E3 ubiquitin-protein ligase and a signal-transducing adaptor protein involved in the development and progression of cancer. Despite the known functions of Cbl-b, its role in breast cancer remains unclear. The aim of this study is to explore the prognostic value of Cbl-b in breast cancer.

**Methods:** Cbl-b expression was analyzed by immunohistochemistry in 292 breast cancer patients from the First Hospital of China Medical University between 1999 and 2008. Kaplan-Meier curve and Cox proportional hazards regression were used to analyze the independent prognostic factors for overall survival (OS) and disease-free survival (DFS). Nomogram was constructed based on these prognostic factors.

**Results:** Cbl-b expression was detected in 54.1% (158/292) breast cancer tissue samples. Cbl-b expression was correlated with DFS (*p* = 0.033), but was not significantly associated with the known clinic-pathological factors in this study. Log-rank analysis indicated that Cbl-b expression was correlated with better OS (*p* = 0.013) and DFS (*p* = 0.016). Multivariate analysis showed that Cbl-b expression was an independent prognostic factor in breast cancer. The nomogram we built for predicting OS was integrated with Cbl-b expression, age, tumor size, lymph node metastasis and histological grade. Except tumor size, all the above factors and date of diagnosis were used to construct the DFS nomogram. The C-indexes of the nomograms were 0.735 and 0.678, respectively. Our new clinical model was superior to the TNM staging for prediction of OS.

**Conclusion:** Cbl-b expression independently predicts favorable prognosis in breast cancer. Cbl-b expression, combined with other variables could be more precise clinical predictive models for predicting OS and DFS in patients with breast cancer.

## Introduction

Breast cancer is the most common malignancy and the leading cause of cancer death amongst women worldwide. It was estimated that there were 2.1 million newly diagnosed female breast cancer cases worldwide in 2018, accounting for a quarter of female cancer cases (1). Exploring the prognostic elements of breast cancer may provide clues for developing potential therapeutic targets. Some clinic-pathological features and biological indicators have been reported as prognostic factors for breast cancer patients, including hormone receptor (HR) status, which refers to estrogen receptor (ER) and progesterone receptor (PR) status, human epidermal growth factor receptor2 (HER2) status, histological grade, pathological type, TNM staging, age at diagnosis, marital status and some tumor markers such as CEA, CA15-3 (2–5). However, exploring new prognostic markers and models has high potential to improve outcomes for patients with breast cancer. Our previous study (6) revealed that Casitas B-lineage lymphoma proto-oncogene-b (Cbl-b) might be a prognostic indicator in breast cancer by regulating the signaling pathways and cell proliferation.

It has been proposed that Cbl-b functions as an E3 ubiquitin protein ligase, thereby negatively regulating receptor tyrosine kinase (RTK) signal-transduction pathways and inhibiting tumor growth (7, 8). In addition, Cbl-b protein could regulate cell activation by acting as an adaptor protein. In the immune system, Cbl-b can play key roles in anti-tumor immunity by regulating T (9), B (10), and NK cell (11) activation pathways.

The prognostic value of Cbl-b in different tumors remains controversial. It has been reported that Cbl-b expression can predict poor prognosis in resectable pancreatic ductal adenocarcinoma (12). Conversely, our team has reported that Cbl-b can act as a favorable prognostic factor in multiple drug resistance gastric cancer (13) and non-small cell lung cancer (14) by inhibiting cancer cell proliferation. Furthermore, a preclinical study (15) has indicated that Cbl-b expression can inhibit breast cancer cell proliferation and migration *in vivo* by decreasing the activation of AKT and ERK. It suggested that Cbl-b may be a favorable prognostic factor. However, the clinical prognostic value of Cbl-b in patients with breast cancer remains unclear.

In the current study, we aimed to explore the prognostic value of Cbl-b expression in breast cancer. Furthermore, we developed new prognostic nomograms based on Cbl-b and other variables for predicting overall survival (OS) and disease-free survival (DFS). This may improve our understanding of the prognostic value of Cbl-b in breast cancer and provide more accurate individual prognosis estimates.

## Materials and Methods

### Patients and Tissue Samples

This study included 292 female patients with breast cancer who received surgery from the First Hospital of China Medical University between January 1999 and December 2008. Inclusion criteria were as follows: clear diagnosis by pathology; complete clinical data and follow-up data; patients with breast cancer surgery; patients who received postoperative adjuvant therapy (including chemotherapy, radiotherapy or endocrine therapy). However, because of the inaccessibility of drugs or unreimbursment by medical insurance in China in 1999–2008, we did not include anti-HER2 targeted therapy as the inclusion criteria. Exclusion criteria included patients with *de novo* stage IV breast cancer; patients who were combined with other malignant tumors.

### Immunohistochemistry

Tumor specimens were collected from the Department of Pathology at the First Hospital of China Medical University. Immunohistochemistry staining for Cbl-b was performed as described previously (16). The immunoreactivity of Cbl-b was scored based on both intensity of staining (negative = 0, weak = 1, moderate = 2, strong = 3) and percentage of positive tumor cells (<10% = 0, 10–50% = 1, >50% = 2). The final score was calculated by multiplying the single scores obtained from the intensity and percentage of positive cells (ranging from 0 to 6) (6). The median expression score of Cbl-b was 2, which could be used as a cut-off value. Then patients with a score of at least 2 being applicable to the Cbl-b positive study population. Two pathologists independently scored the slides.

### Statistical Analysis

Data analysis was performed using SPSS software version 19.0 and R 3.6.0. The correlation between Cbl-b expression and clinic-pathological variables of breast cancer patients was assessed using the Chi-square test. Kaplan-Meier method was adopted to map survival curves of patients with breast cancer. Univariate and multivariate analyses were performed according to the Cox proportional hazards model. Statistically significant variables (*P* < 0.05) in multivariate analysis were included in the final nomograms. The C-index was used to evaluate the discrimination of the nomogram. The higher the C-index, the more accurate the prognostic prediction was. The receiver operating characteristic (ROC) analysis was also used to verify the accuracy of our new prognostic model.

## Results

### Demographic and Clinic-Pathological Characteristics

A total of 292 histologically confirmed breast cancer samples between January 1999 and December 2008 were obtained. The majority of patients (88.7%) were diagnosed with invasive ductal carcinoma (*n* = 259). The median age of patients was 51 years (ranging from 26 to 76 years), and the median follow-up time was 125.7 months (ranging from 15.7 to 208 months). During the follow-up period, 99 patients developed disease progression (33.9%), of which 69 patients died of disease progression (23.6%).

**Figure 1:**
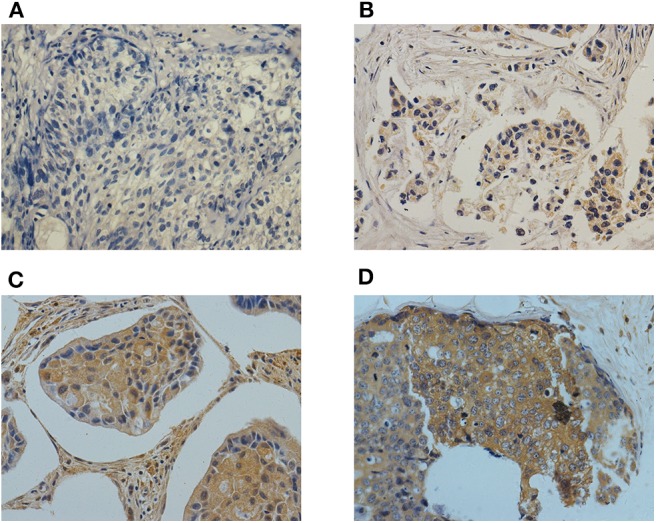
Representative images of Cbl-b immunohistochemical staining for each of the scores (0–3) in breast cancer tissues. Magnification × 400. **(A)** Negetive control (score = 0), **(B)** Cbl-b weak positive (score = 1), **(C)** Cbl-b moderate positive (score = 2), **(D)** Cbl-b strong positive (score = 3).

Immunohistochemistry was performed to evaluate Cbl-b expression. Positive Cbl-b expression was observed in 54.1% (158/292) breast cancer tissue samples, Cbl-b staining for each of the scores (0–3) was shown in Figure 1. Cbl-b expression was significantly correlated with DFS (*p* = 0.033), but was not associated with HR or HER2 status, and other clinic-pathological factors (*p* > 0.05, Table 1).

**Table 1:** Correlation of Cbl-b expression with clinic-pathological parameters in patients with breast cancer.

	***n***	**Cbl-b negative (*n*, %)**	**Cbl-b positive (*n*, %)**	***P*-value**
Age (years)				0.556
>35	279	127 (94.8)	152 (96.2)	
≤ 35	13	7 (5.2)	6 (3.8)	
Pathological type				0.501
Invasive ductal carcinomas	259	116 (86.6)	143 (90.5)	
Invasive lobular carcinomas	17	10 (7.5)	7 (4.4)	
Others	16	8 (6.0)	8 (5.1)	
Histology grade				0.774
1 or unknown^a^	61	27 (20.1)	34 (21.5)	
2–3	231	107 (79.9)	124 (78.5)	
Tumor size (cm)				0.098
≤ 2	95	37 (27.6)	58 (36.7)	
>2	197	97 (72.4)	100 (63.3)	
Lymph node metastasis				0.746
No	125	56 (41.8)	69 (43.7)	
Yes	167	78 (58.2)	89 (56.3)	
Stage				0.188
0–I	53	20 (14.9)	33 (20.9)	
II–III	239	114 (85.1)	125 (79.1)	
ER status				0.424
Negative	161	74 (55.2)	87 (55.1)	
Positive	129	60 (44.8)	69 (43.7)	
Unknown	2	0 (0)	2 (1.3)	
PR status				0.404
Negative	155	73 (54.5)	82 (51.9)	
Positive	135	61 (45.5)	74 (46.8)	
Unknown	2	0 (0)	2 (1.3)	
HR status				0.405
Negative	127	60 (44.8)	67 (42.4)	
Positive	163	74 (55.2)	89 (56.3)	
Unknown	2	0 (0)	2 (1.3)	
HER2 status				0.667
Negative	185	87 (64.9)	98 (62.0)	
Positive	102	44 (32.8)	58 (36.7)	
unknown	5	3 (2.2)	2 (1.3)	
HR status and HER2 status				0.719
HR positive HER2 negative	113	50 (37.3)	63 (39.9)	
HR positive HER2 positive	46	22 (16.4)	24 (15.2)	
Triple negative	71	37 (27.6)	34 (21.5)	
HR negative HER2 positive	55	22 (16.4)	33 (20.9)	
Unknown	7	3 (2.2)	4 (2.5)	
Menstrual status				0.951
Premenopausal	164	75 (56.0)	89 (56.3)	
Postmenopausal	128	59 (44.0)	69 (43.7)	
Surgery type				0.423
Radical mastectomy	288	133 (99.3)	155 (98.1)	
Breast-conserving surgery	2	1 (0.7)	1 (0.6)	
Simple mastectomy	2	0 (0)	2 (1.3)	
Neoadjuvant chemotherapy				0.17
No	285	129 (96.3)	156 (98.7)	
Yes	7	5 (3.7)	2 (1.3)	
Adjuvant chemotherapy				0.331
Anthracycline-based only	166	69 (51.5)	97 (61.4)	
Taxane-based only	5	3 (2.2)	2 (1.3)	
Anthracycline & taxane-based	101	53 (39.6)	48 (30.4)	
Others^b^	20	9 (6.7)	11 (7.0)	
Endocrine therapy				0.85
No	129	60 (44.8)	69 (43.7)	
Yes	163	74 (55.2)	89 (56.3)	
Radiotherapy				0.935
No	199	91 (67.9)	108 (68.4)	
Yes	93	43 (32.1)	50 (31.6)	
Trastuzumab therapy				0.352
No	285	132 (98.5)	153 (96.8)	
Yes	7	2 (1.5)	5 (3.2)	
Date of diagnosis				0.631
1999–2003	109	52 (38.8)	57 (36.1)	
2004–2008	183	82 (61.2)	101 (63.9)	
DFS (months)				**0.033**
≤ 12	13	6 (4.5)	7 (4.4)	
13–36	43	22 (16.4)	21 (13.3)	
37–120	92	52 (38.8)	40 (25.3)	
>120	144	54 (40.3)	90 (57.0)	
Age (years)				0.269
≤ 50	141	60 (44.8)	81 (51.3)	
>50	151	74 (55.2)	77 (48.7)	

*P value shown in bold is statistically significant (two-sided, p < 0.05)*.

a *There is a small percentage of missing data about histology grade, which is not more than 10%*.

b *The group contained patients who received CMF, xeloda, NP or GP*.

### Relationship Between Cbl-b Expression and Prognosis of Patients With Breast Cancer

**Figure 2:**
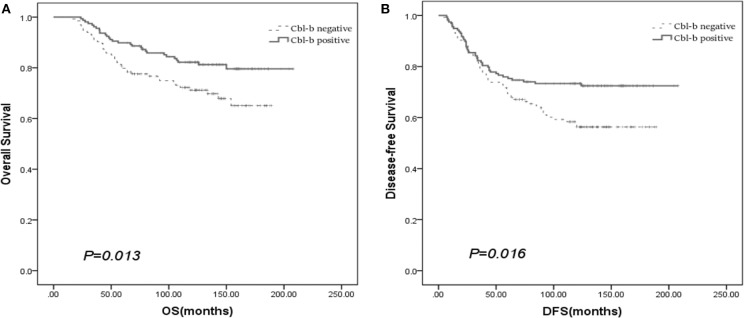
The prognostic value of Cbl-b in patients with breast cancer for OS **(A)** and DFS **(B)**.

We evaluated the prognostic value of Cbl-b in patients with breast cancer through survival analyses. Kaplan–Meier survival analyses revealed that Cbl-b expression was significantly associated with better OS (*p* = 0.013) and DFS (*p* = 0.016) in patients with breast cancer, though the median OS and DFS were not reached at the last follow-up of November 2017 (Figures 2A,B).

**Figure 3:**
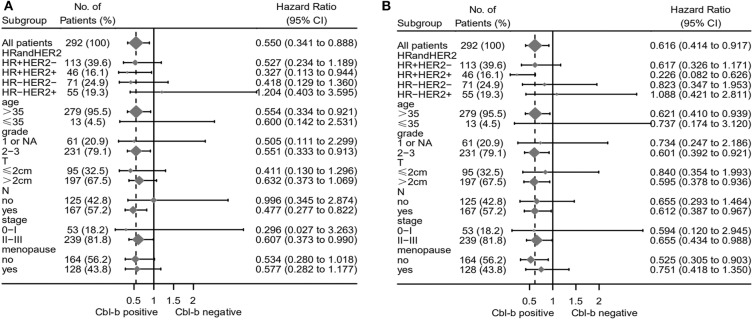
Forest plot of subgroup analysis OS **(A)** and DFS **(B)** for patients with breast cancer.

The forest plot of subgroup analysis showed that positive Cbl-b expression was significantly associated with favorable prognosis in terms of OS in patients with HR-positive/HER2-positive, age > 35 years, histological grade 2-3, lymph nodes metastasis, and stage II-III breast cancer (Figure 3A). Moreover, positive Cbl-b expression indicated favorable prognosis in terms of DFS for patients with HR-positive/HER2-positive, age > 35 years, histological grade 2–3, tumor size > 2 cm, lymph node metastasis, stage II–III and premenopausal breast cancer (Figure 3B).

Univariate survival analysis showed that the patients with Cbl-b expression, age, tumor size, lymph node metastasis, histological grade, stage, radiotherapy and trastuzumab therapy were associated with prognosis (*p* < 0.2). In addition, HER2 status and neoadjuvant chemotherapy were also correlated with OS. And date of diagnosis was associated with DFS. Though HR status and HER2 status did not show statistical significance, many studies have confirmed their clinical importance. So we incorporated all the above factors into multivariate survival analysis. The results indicated that Cbl-b expression, age, tumor size, lymph node metastasis and histological grade were independent predictors for OS (Table 2A). Besides, Cbl-b expression, age, lymph node metastasis, histological grade and date of diagnosis were all independently associated with DFS (Table 2B).

**Table 2:** Cox univariate and multivariate analysis of OS (A) and DFS (B) in patients with breast cancer.

**Risk factors**	**Univariate analysis**		**Multivariate analysis**		**Score**
	**HR (95%CI)**	***P*****-value**	**HR (95%CI)**	***P*****-value**	
**A**					
**Cbl-b**					
Positive	1		1		0
Negative	1.817 (1.126–2.933)	**0.014**	1.705 (1.054–2.759)	**0.03**	1
**Age (years)**					
>35	1		1		0
≤ 35	4.105 (1.961–8.592)	**<0.001**	3.922 (1.863–8.254)	**<0.001**	2
**Tumor size**					
≤ 2 cm	1				0
>2 cm	2.504 (1.343–4.667)	**0.004**	1.896 (1.008–3.565)	**0.047**	1
**Lymph node metastasis**					
No	1		1		0
Yes	3.371 (1.874–6.064)	**<0.001**	2.874 (1.588–5.202)	**<0.001**	2
**Histology grade**					
1 or unknown	1		1		0
2–3	2.843 (1.300–6.215)	**0.009**	2.376 (1.081–5.220)	**0.031**	1
**Stage**					
0–I	1				
II–III	5.430 (1.707–17.270)	**0.004**			
**HR**					
Negative	1				
Positive	1.138 (0.702–1.846)	0.6			
**HER2**					
Negative	1				
Positive	1.612 (0.996–2.610)	**0.052**			
**Date of diagnosis**					
2004–2008	1				
1999–2003	1.307 (0.808–2.114)	0.276			
**Neoadjuvant chemotherapy**					
No	1				
Yes	2.147 (0.675–6.834)	**0.196**			
**Adjuvant chemotherapy**		0.357			
Anthracycline-based only	1				
Taxane-based only	0.978 (0.134–7.141)	0.982			
Anthracycline & taxane-based	1.309 (0.783–2.189)	0.304			
Others	1.941 (0.900–4.186)	0.091			
**Endocrine therapy**					
No	1				
Yes	1.161 (0.715–1.883)	0.546			
**Radiotherapy**					
No	1				
Yes	1.937 (1.207–3.109)	**0.006**			
**Trastuzumab therapy**					
No	1				
Yes	2.374 (0.746–7.556)	**0.143**			
**Surgery type**		0.841			
Radical mastectomy	1				
Breast-conserving surgery	0	0.974			
Simple mastectomy	1.806 (0.251–13.012)	0.557			
**Menstrual status**					
Premenopausal	1				
Postmenopausal	1.006 (0.626–1.617)	0.982			
**Age (years)**					
≤ 50	1				
>50	0.997 (0.621–1.599)	0.989			
**B**					
**Cbl-b**					
Positive	1		1		0
Negative	1.623 (1.091–2.416)	**0.017**	1.612 (1.082–2.402)	**0.019**	1
**Age (years)**					
>35	1		1		0
≤ 35	2.517 (1.221–5.188)	**0.012**	2.980 (1.406–6.315)	**0.004**	2
**Tumor size**					
≤ 2cm	1				
>2 cm	1.965 (1.214–3.183)	**0.006**			
**Lymph node metastasis**					
No	1		1		0
Yes	2.741 (1.730–4.342)	**<** **0.001**	2.569 (1.619–4.076)	**<** **0.001**	2
**Histology grade**					
1 or unknown	1		1		0
2–3	2.050 (1.144–3.674)	**0.016**	1.933 (1.075–3.477)	**0.028**	1
**Stage**					
0–I	1				
II–III	3.915 (1.714–8.941)	**0.001**			
**HR**					
Negative	1				
Positive	1.131 (0.756–1.693)	0.55			
**HER2**					
Negative	1				
Positive	1.251 (0.832–1.881)	0.281			
**Date of diagnosis**					
2004–2008	1		1		0
1999–2003	1.691 (1.137–2.514)	**0.009**	1.905 (1.262–2.876)	**0.002**	1
**Neoadjuvant chemotherapy**					
No	1				
Yes	1.310 (0.415–4.134)	0.645			
**Adjuvant chemotherapy**		0.562			
Anthracycline-based	1				
Taxane-based	0.563 (0.078–4.072)	0.569			
Anthracycline & taxane-based	1.303 (0.858–1.981)	0.215			
Others	1.198 (0.569–2.523)	0.634			
**Endocrine therapy**					
No	1				
Yes	1.154 (0.771–1.728)	0.486			
**Radiotherapy**					
No	1				
Yes	1.801 (1.209–2.683)	**0.004**			
**Trastuzumab therapy**					
No	1				
Yes	2.079 (0.764–5.653)	**0.152**			
**Surgery type**		0.839			
Radical mastectomy	1				
Breast–conserving surgery	0	0.968			
Simple mastectomy	1.812 (0.253–13.005)	0.554			
**Menstrual status**					
Premenopausal	1				
Postmenopausal	1.035 (0.697–1.538)	0.864			
**Age (years)**					
≤ 50	1				
>50	1.014 (0.683–1.504)	0.946			

*P value shown in bold are statistically significant (two-sided, p < 0.2 in univariate analysis and p < 0.05 in multivariate analysis)*.

*The score of each prognostic factor was calculated based on the ratio of their HR values (the score of the least HR value was set as 1)*.

### Predictive Nomograms for OS and DFS

**Figure 4:**
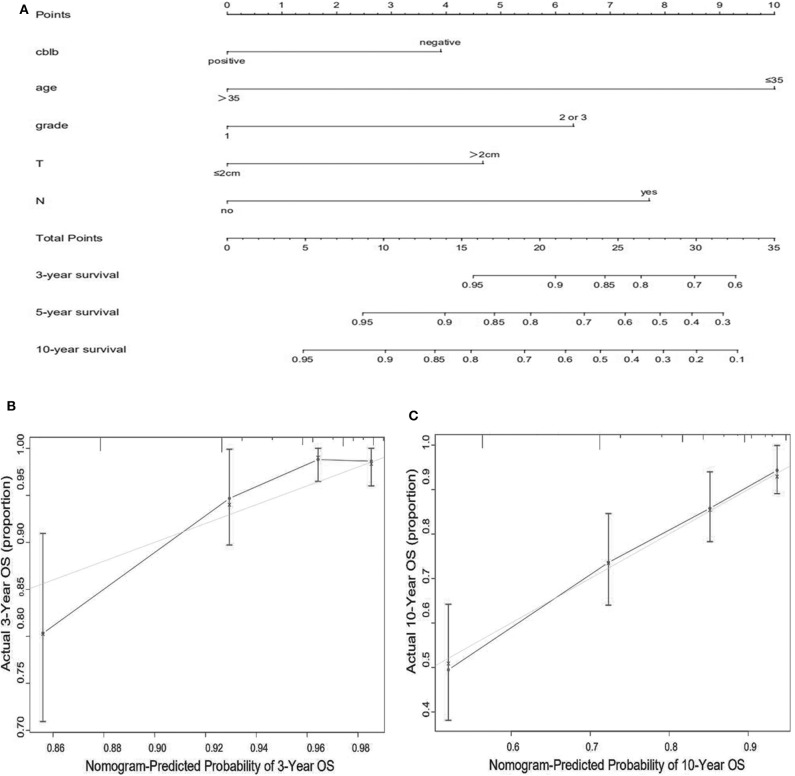
Prognostic nomogram **(A)** and calibration curve **(B,C)** for predicting OS in patients with breast cancer.

**Figure 5:**
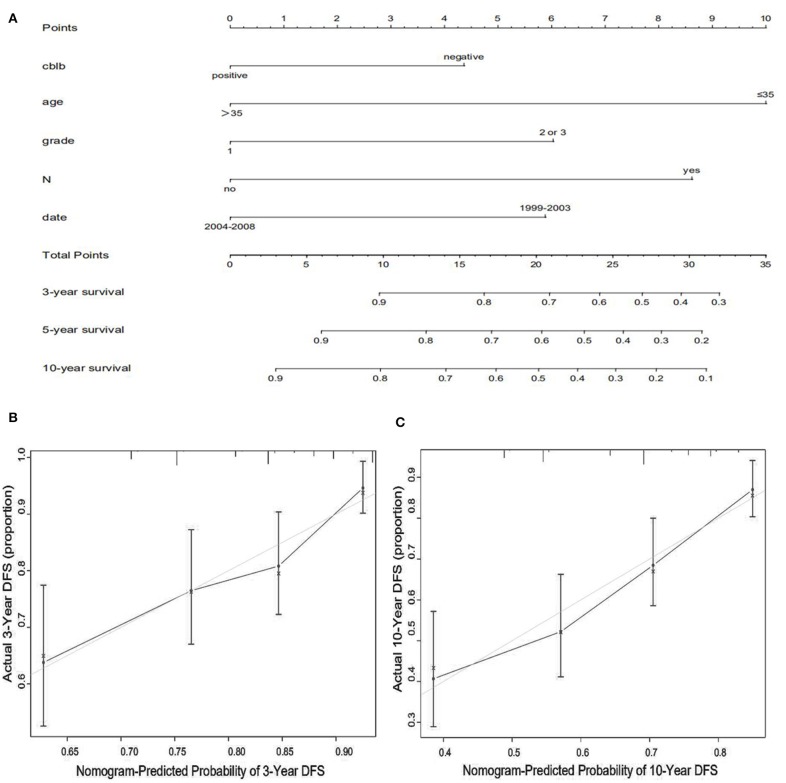
Prognostic nomogram **(A)** and calibration curve **(B,C)** for predicting DFS in patients with breast cancer.

We constructed predictive nomograms based on the multivariate analysis using a cut-off value of *P* < 0.05. According to the aforementioned multivariate survival analysis, the prognosis of patients with breast cancer could be predicted by using this model. Each variable in the nomogram was weighted based on some scores. We then obtained the total score of predicting 3- and 10-year OS, and DFS for each patient. The higher the scores patients obtained, the worse the predicted result. The nomogram was accurate for predicting OS, with Harrell's concordance index (C-index) of 0.735 [95% confidence interval (CI): 0.678–0.792] (Figure 4A). And the C-index of the nomogram for predicting DFS was 0.678 (95% CI:0.628–0.728) (Figure 5A). Calibration curves for predicting 3- and 10-year OS (Figures 4B,C) and DFS (Figures 5B,C) of the nomograms were consistent with the models.

### Different Risk Subgroups Identified According to a Prognostic Score Index

**Figure 6:**
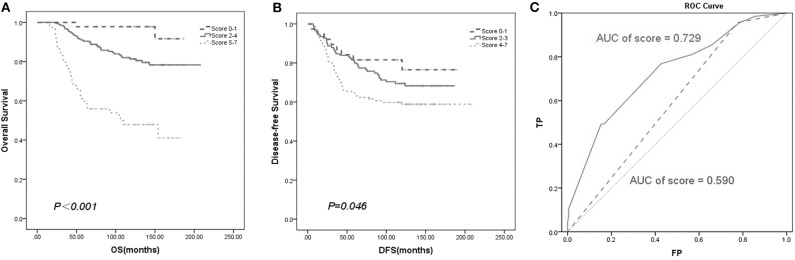
Survival curve based on prognostic score system. The new prognostic factors have separated the patients into different risk subgroups for OS **(A)** and DFS **(B)**. **(C)** ROC analysis of overall survival rate based on established model (AUC of score = 0.729) and TNM stage (AUC of stage = 0.590) for breast cancer patients (*n* = 292).

We established a new prognostic score system to predict patient survival. The score of each prognostic factor was calculated based on the ratio of their HR values gained from the multivariate analysis (Tables 2A,B). Then, we summed the score of the above variables to develop a prognostic score index. Finally, Kaplan–Meier survival analysis of the prognostic score index showed that all the patients with breast cancer could be separated into different risk subgroups for OS with scores ranging from 0–1, 2–4, and 5–7 (Figure 6A). Similarly, the different risk subgroups for DFS were scored 0–1, 2–3, and 4–7 (Figure 6B). Patients with a higher score were predicted to have poorer prognosis.

To verify the accuracy of our new prognostic model compared with the TNM staging prognostic model, ROC analysis was performed. Our model yielded an area under the receiver operating characteristic curve (AUC) of 0.729 for predicting OS, which was superior to TNM staging according to the AJCC 8th with an AUC of 0.590 (Figure 6C).

## Discussion

Breast cancer is a highly heterogeneous disease. Several previous studies (17, 18) have summarized the prognostic factors and survival outcomes associated with breast cancer. Our results indicated that positive Cbl-b expression was an independent favorable prognostic factor in breast cancer. To the best of our knowledge, this is the first study to build nomograms based on Cbl-b and other variables that can be used to predict prognosis of patients with breast cancer. The novel prognostic model could improve the accuracy of traditional TNM staging for predicting OS.

In the present study, we firstly reported that Cbl-b was expressed in 54.1% (158/292) of breast cancer tissues. Our previous studies also showed that positive Cbl-b expression was observed in 69.5% (107/154) receptor activator of nuclear factor κ-B (RANK) positive breast cancer samples (6), and 60.3% (73/121) patients with P-glycoprotein positive multiple drug resistance breast cancer (16).

The E3 ubiquitin-protein ligase Cbl-b may have controversial functions in different tumor types. Cbl-b was reported to function as a negative regulator of growth factor receptor signaling and could suppress tumor cell proliferation (15). Therefore, Cbl-b might have anti-tumor activity. We have found that Cbl-b could inhibit cell proliferation by ubiquitinating the survival signal of the epidermal growth factor receptor (EGFR) pathway in lung (19, 20) and gastric cancer cells (13, 21, 22). Also, previous studies showed that Cbl-b could inhibit tumor growth in some breast cancer cells (6, 23). However, in other tumor types Cbl-b may promote the migration and proliferation of cancer cells. The expression of Cbl-b in skull base chordomas (24) and resectable pancreatic ductal adenocarcinoma (25) can predict tumor invasion and poor prognosis. Overall, the diversity of substrate proteins and cell type-dependent regulation of Cbl-b ubiquitination in tumor cells may explain why Cbl-b displays differential functions in different tumor cells. Our study suggested that Cbl-b expression was an independent prognostic factor. In addition, in the subgroup analysis we found that positive Cbl-b expression indicated favorable prognosis mainly in patients with histological grade 2–3, lymph node metastasis, and stage II–III. It was worth noting that all the above subgroups were relatively poor prognostic, indicating that the protective effect of Cbl-b might occur in clinically recognized subgroups of poor prognosis patients.

Recently it has been reported that a series of nomograms were built to predict prognosis of breast cancer. Xiong et al. (26) developed a nomogram to predict OS of metastatic breast cancer with the following factors: age, metastasis-free interval, metastasis location, and HR status. The C-index value of this model was 0.69. Prognostic factors used to create the nomograms of triple-negative breast cancer (27) and young women with breast cancer (28) included race, tumor size, number of positive lymph nodes, grade, and histological sub-type. The C-indexes for predicting OS were 0.779 and 0.724, respectively. To our knowledge, our study was the first to construct comprehensive nomograms for predicting both OS and DFS based on Cbl-b expression, age, lymph node metastasis, histological grade and other variables in breast cancer. The C-indexes of our nomograms were 0.735 for OS and 0.678 for DFS, indicting that our model showed good accuracy for predicting OS but was a weak model for DFS. Though the accuracy of DFS nomogram needed to be further improved, combining Cbl-b expression with other variables may provide a more precise prognostic model than traditional TNM stage system. Therefore, Cbl-b is still a critical prognostic factor in breast cancer.

The present study has some limitations. Firstly, we did not provide Ki67 and subsequent retesting or treatments. So we could not provide the precise luminal type, which is important for the choice of treatment and prognosis of breast cancer. Secondly, HR-positive patients accounted for only 55.8% of 292 patients with breast cancer, thus the distribution of tumor sub-type in our study could not stand for usual distribution. In addition, most HER2-positive patients did not receive trastuzumab due to the inaccessibility of drugs or unreimbursment by medical insurance in China in 1999–2008. Lastly, this study was performed retrospectively and requires larger multi-institutional prospective studies to further validate our findings in the future.

In conclusion, the expression of Cbl-b might be viewed as an independent prognostic factor for patients with breast cancer. Combining Cbl-b expression with other variables may provide a more precise prognostic model for patients with breast cancer. Although targeted drugs for Cbl-b have not yet been developed, the development of ubiquitin ligases (E3s) inhibitors are already being tested in clinical trials phase I, including inhibitors of MDM2, SPOP, and cIAP (29). Therefore, detection of Cbl-b expression may help clinicians make accurate individual prognosis estimates and provide clues for developing potential therapeutic targets for patients with breast cancer. However, our results are just hypothesis generating and further assessment in prospective trials are needed in order to evaluate the prognostic role of Cbl-b.

## Data Availability Statement

The datasets generated for this study are available on request to the corresponding author.

## Ethics Statement

The studies involving human participants were reviewed and approved by Ethics Review Board of The First Hospital of China Medical University. Written informed consent for participation was not required for this study in accordance with the national legislation and the institutional requirements.

## Author Contributions

LZ and XL designed the study and collected data. XL wrote the manuscript. XL, XW, BB, and ZL contributed in the statistical analysis.YT, XQ, YL, and LZ supervised and approved the final draft.

## Conflict of Interest

The authors declare that the research was conducted in the absence of any commercial or financial relationships that could be construed as a potential conflict of interest.
